# Cigarette Litter: Smokers’ Attitudes and Behaviors

**DOI:** 10.3390/ijerph9062189

**Published:** 2012-06-13

**Authors:** Jessica M. Rath, Rebecca A. Rubenstein, Laurel E. Curry, Sarah E. Shank, Julia C. Cartwright

**Affiliations:** 1 Research & Evaluation Department, Legacy, 1724 Massachusetts Avenue NW, Washington, DC 20036, USA; Email: brubenstein@legacyforhealth.org (R.A.R.); lcurry@rti.org (L.E.C.); 2 Communications Department, Legacy, 1724 Massachusetts Avenue NW, Washington, DC 20036, USA; Email: sshank@legacyforhealth.org (S.E.S.); jcartwright@legacyforhealth.org (J.C.C.)

**Keywords:** tobacco, litter, environment, cigarette filters, cigarette butt trash, butt waste

## Abstract

Cigarette butts are consistently the most collected items in litter clean-up efforts, which are a costly burden to local economies. In addition, tobacco waste may be detrimental to our natural environment. The tobacco industry has conducted or funded numerous studies on smokers’ littering knowledge and behavior, however, non-industry sponsored research is rare. We sought to examine whether demographics and smokers’ knowledge and beliefs toward cigarette waste as litter predicts littering behavior. Smokers aged 18 and older (n = 1,000) were interviewed about their knowledge and beliefs towards cigarette waste as litter. Respondents were members of the Research Now panel, an online panel of over three million respondents in the United States. Multivariate logistic regressions were conducted to determine factors significantly predictive of ever having littered cigarette butts or having littered cigarette butts within the past month (*p*-value < 0.05). The majority (74.1%) of smokers reported having littered cigarette butts at least once in their life, by disposing of them on the ground or throwing them out of a car window. Over half (55.7%) reported disposing of cigarette butts on the ground, in a sewer/gutter, or down a drain in the past month. Those who did not consider cigarette butts to be litter were over three and half times as likely to report having ever littered cigarette butts (OR = 3.68, 95%CI = 2.04, 6.66) and four times as likely to have littered cigarette butts in the past month (OR = 4.00, 95%CI = 2.53, 6.32). Males were significantly more likely to have littered cigarette butts in the past month compared to females (OR = 1.49, 95%CI = 1.14, 1.94). Holding the belief that cigarette butts are not litter was the only belief in this study that predicted ever or past-month littering of cigarette waste. Messages in anti-cigarette-litter campaigns should emphasize that cigarette butts are not just litter but are toxic waste and are harmful when disposed of improperly.

## 1. Introduction

In addition to being the primary cause of preventable death in the United States (US), smoking is currently the leading contributor to the nation’s litter burden [1,2]; cigarette butts are consistently the most littered item in the U.S. In 2009, an estimated 51.2 billion pieces of litter were recovered from roadways in the U.S., of which 38% were tobacco products [3]. Cigarette butts were also the most collected items during clean-up efforts in other locations, including retail areas, storm drains, loading docks, construction sites, parks and playgrounds [3]. Along beaches and waterways, cigarettes were the number one debris item picked up over the past 25 years during the Ocean Conservancy’s International Coastal Cleanup (ICC) [4]. Tobacco product litter cleanup is costly: an economic analysis found that cities the size of San Francisco spend, on average, between $500,000 and $6 million annually to keep their streets and parks clear of cigarette litter [5]. 

Limited research shows that cigarette butts do not biodegrade under typical circumstances [6,7]. Cigarette filters are made of cellulose acetate, a plastic containing naturally occurring cellulose modified by adding acetate compounds. Under certain conditions, cellulose acetate is photodegradable and biodegradable [6,8,9,10]. However, because the plastic in cigarette filters contains particularly high amounts of acetyl groups and is tightly compressed, cigarette butts only disintegrate under conditions described as “severe biological circumstances”, such as submersion in sewage [6,8,11]. Very few studies have examined the biodegradability of cigarette butts and most were sponsored by the tobacco industry during a period when they explored manufacturing a biodegradable filter [12]. In 1991, scientists hired by British American Tobacco tested how cigarette filters degraded under various environmental conditions and found that cigarette butts left on a city pavement showed no indication of degrading over two months [6]. Although the tobacco industry has notoriously misrepresented research findings [13], these particular studies were only recently widely available through the Legacy Tobacco Documents Library and were commissioned by the tobacco industry specifically to make internal business decisions [12]. Because these memos were never intended to be public, we can reasonably rely on the results. 

Preliminary research suggests that pervasive tobacco waste may be detrimental to our natural environment. Chemicals released from remnants of the tobacco in cigarette butts have the potential to pollute aquatic ecosystems and harm organisms [14,15,16]. Both smoked and un-smoked cigarettes demonstrate the potential for rapid and prolonged metal contamination of the immediate environment in which they are discarded: in a laboratory study, both released numerous heavy metals into water [15]. Likewise, other research found that one cigarette butt soaked in a liter of water killed half of the fish exposed [14]. 

A review of tobacco industry internal documents revealed that, since the 1970s, companies have feared the tobacco litter issue might lead to regulation or affect the social acceptability of smoking [12]. One memo cites the perception of the smoker as “careless, offensive and occasionally harmful with his debris” as one cause of the “growing social disapproval of…smoking” [17]. In turn, tobacco companies have teamed up with anti-litter groups, particularly Keep America Beautiful, a national non-profit, in order to carefully steer anti-litter efforts in their favor [18,19]. Similarly, the United Kingdom’s Tobacco Manufacturers Association (TMA) boasts that it works closely with Britain’s national anti-litter group, Keep Britain Tidy (formerly Environmental Campaigns or ENCAMS) which has directed several anti-litter campaigns [20,21,22]. Although Keep Britain Tidy receives much of its funding from government agencies [23], they formed the Cigarette Litter Action Group in 2007 “to deal with the growing problem of cigarette litter” and included TMA members on the committee. TMA’s members include British American Tobacco UK Ltd., Gallaher Ltd., and Imperial Tobacco Ltd. [24].

In addition to sponsoring anti-litter campaigns, tobacco companies or industry-funded anti-litter groups have conducted virtually all research on smokers’ litter attitudes and behavior. In the 1990s, tobacco companies studied smokers’ litter beliefs and behaviors through internally funded focus groups and interviews in order to craft anti-littering messages that were neither anti-smoking nor anti-smoker [12,19]. Like the cigarette butt biodegradability reports, this market research was designed to truly uncover smokers’ attitudes on cigarette butt litter. For this reason, our study relies on formerly secret industry data for both background information and for comparison to our own findings. However, this data is outdated and to the extent the industry continues to conduct similar research, it is not publically available. Therefore, new, equally unbiased studies on tobacco litter attitudes and beliefs are needed. 

Other than a handful of state or local reports, more recent cigarette litter attitude survey research is conducted by tobacco industry supported non-profit groups. For instance, Keep America Beautiful’s three-pronged 2009 Littering Behavior in America research, self-described as the “largest litter study ever conducted in the United States”, and by the organization as the “first major national litter study in 40 years”, was funded by Philip Morris USA [25,26]. Tobacco industry sponsored anti-litter research reports intended for public consumption may be inherently biased because the industry’s priority to retain customers supersedes any sincere effort to fully understand why smokers continue to litter despite years of anti-litter campaigns. These conflicting goals may partially explain why industry funded anti-tobacco litter campaigns have failed to show a reduction in tobacco litter [19,27]. The 2009 Littering Behavior in America telephone survey does not examine knowledge or attitudes about the toxicity of cigarette waste nor does it include items about dependence [28] arguably because the industry does not want to draw attention to either issue although both may influence littering. Thus, research without an industry bias is crucial to begin consciously addressing the issue of why tobacco waste persists. 

This non-industry supported, national study examines to what extent smokers’ beliefs toward tobacco products as litter affected their littering behavior recently and in their lifetimes and whether demographics or beliefs were associated with littering behavior. 

## 2. Methodology

In April 2011, smokers and nonsmokers aged 18 and older (n = 2,000) from four cities and a national control sample were interviewed about their smoking patterns and knowledge and beliefs toward cigarette waste as litter. The survey was conducted to evaluate a communications initiative about the environmental impact of cigarette butts targeting these cities. The pre-communications-initiative sample was used for this analysis. Respondents were members of the Research Now panel, an online panel of over three million participants across the U.S. Multivariate logistic regressions were constructed to determine the factors that were significantly predictive of past-month or ever littering (*p*-value < 0.05). This study was approved by the Independent Institutional Review Board, Inc.

### 2.1. Sample

Two hundred smokers and 200 nonsmokers (18 years and older) were drawn from each of four cities. In addition, a sample (n = 400) was collected from designated media areas (DMAs) outside of the four cities targeted to receive the campaign message. The survey was originally developed to evaluate Legacy’s media campaign scheduled around Earth Day 2011, called “Butt Really”. While the results of the “Butt Really” campaign were null for demonstrating an increase in campaign awareness in selected cities, trends were in the right direction in terms of changing knowledge, attitudes, beliefs and behaviors regarding the toxic impact cigarette butts may have on the environment. Forty four percent of survey indicators changed at least 1% in the right direction from pretest (before campaign launch) to posttest (after campaign launch) for the intervention cities. The campaign was the recipient of multiple awards for its creativity and design to raise public awareness surrounding the toxic impact that cigarette butts have on the environment by harnessing both mass media and social media strategies [29,30,31]. This study is a secondary analysis using data from an evaluation of the “Butt Really” campaign. The sample was drawn from cities with relatively large media markets, to maximize the possibility of detecting a media effect. In addition, cities were selected that had larger totals of cigarette litter from the 2010 ICC report [4], to increase the odds that the “Butt Really” message would resonate with the target audience. A total of 2,000 participants were randomly sampled from members of the Research Now online panel in Atlanta, Houston, Baltimore, and Charlotte. Panelists are adults aged 18 and older, recruited through email and online with over 300 diverse online affiliate partners and targeted website advertising. Immediately upon completion of the survey, virtual currency is awarded to each respondent which can be combined with virtual currency from other surveys and exchanged for rewards. The virtual currency incentive for this survey was equivalent to $4.

### 2.2. Measures

The survey instrument covered four domains: demographics (age, zipcode, gender, race/ethnicity, and education); smoking status; knowledge, beliefs and behaviors regarding cigarette litter; and awareness of the media campaign. The survey questions were originally designed to measure awareness of the Butt Really campaign. We surveyed two groups of respondents, one before and one after the campaign was launched. The “past-30 day” questions were designed to capture whether participants were aware of the exact talking points of the campaign. The “ever litter” questions, on the other hand, were designed to gauge general “ever littering” behavior among former and current smokers in the sample.

*Smoking status*. Smoking status questions were measured by items from the National Health Interview Survey, 2011 and the National Youth Tobacco Survey, 2009 [32,33]. Current smokers were defined as currently smoking cigarettes every day or some days [33] and having smoked at least 1 cigarette in the past 30 days [33]. Former smokers were defined as having smoked at least 100 cigarettes in a lifetime [33] and currently not smoking at all; never smokers were defined as having never tried smoking, even one or two puffs [32]. Average number of cigarettes smoked per day was also collected on a continuous scale [33]. 

*Knowledge and beliefs about cigarette litter*. The knowledge questions regarding cigarette litter were developed by the principal investigator based on talking points from the “Butt Really” campaign and were designed to measure knowledge about the biodegradability of cigarette butts, and whether discarded filters are harmful to humans and animals. Questions included, “the single most collected item in beach waste cleanup each year is?” The four possible response choices included: (1) beverage containers; (2) cigarette filters (butts); (3) fishing line/nets; (4) plastic bags. A set of belief questions was developed regarding cigarette filters to be answered on a 4-point Likert scale from 1 “strongly agree” to 4 “strongly disagree.” Questions included items measuring whether or not respondents considered cigarette butts to be toxic, biodegradable, harmless when eaten by humans, harmless when eaten by animals/marine life, and dangerous if thrown in a trashcan. Responses for each question ranged from 1–4 with lower scores indicating stronger anti-litter beliefs (reverse code biodegradability question). Finally, respondents were asked, “do you consider cigarette butts to be litter?” (yes/no). Awareness questions about the “Butt Really” campaign were also included on the survey but not used in this analysis. 

*Behaviors regarding cigarette litter*. The behavior questions regarding cigarette filters were developed by the principal investigator based on talking points from “Butt Really” to measure behaviors surrounding disposing of cigarette filters/butts. Ever littering of cigarette butts was defined as answering “yes” to at least one of two items: “Many people smoking outdoors dispose of their cigarette butts by dropping them on the ground. Did you ever dispose of your cigarettes in this way?” and “Many people smoking in their cars dispose of their cigarette butts by throwing/dropping them out the car window. Did you ever dispose of your cigarettes in this way?” Past-month littering was defined as selecting “on the ground,” “in the sewer/gutter,” or “down a drain” in response to the item, “In the past 30 days, have you disposed of your cigarette butts in any of these places?” Those who selected “a trashcan,” “public ash tray,” or “personal pocket ashtray” were not considered past-month litterers. 

### 2.3. Statistical Analysis

Only the baseline survey (n = 2,000) was used in this analysis. Two sets of bivariate logistic regressions were utilized to examine the relationship between past-month littering and ever littering as the dependent variables and each of the independent variables (namely, demographics and beliefs about cigarette butts as litter). The multivariate logistic regressions were constructed to determine the factors that were predictive of past-month or ever littering. All variables significantly related (*p*-value < 0.05) to past-month or ever littering in the bivariate logistic regressions were included in the multivariate logistic regression model. A stepwise regression was then used to refine the model, and a third model including all variables, regardless of bivariate significance, was also run to verify results. Results varied only slightly between models as explained below. All statistical analyses were performed using Stata statistical software, version 11.2 [34]. 

**Table 1:** Sociodemographic and smoking characteristics (n = 2,000).

Age	N	%
18–24	106	5.30
25–44	914	45.7
45–64	849	42.5
65+	131	6.6
**Gender**		
Male	866	43.3
Female	1,134	56.7
**Ethnicity**		
Hispanic	116	5.8
Non-Hispanic	1,853	92.7
Decline	31	1.6
**Race**		
White	1,640	82.0
Black/AA	141	7.1
Asian or PI	113	5.7
NA/AN	12	0.60
Mixed Race	51	2.6
Declined	43	2.2
**Education**		
Less than High school	9	0.5
High school	169	8.5
GED	19	1.0
Some College	515	25.8
2-year College	182	9.1
4-year College	689	34.5
Advanced Degree	417	20.9
**Smoking Status **		
Current	1,000	50.0
Never	617	30.9
Former	383	19.2
**Cigarettes per day (smokers)**		
1–20	781	78.1
21+	219	21.9

## 3. Results

Sociodemographic and smoking information about the study population are presented in Table 1. Most of the respondents (n = 2,000) were white (82.0%) and non-Hispanic (92.7%). There were slightly more female respondents (56.7%) than male respondents (43.4%), and the majority were between 25–64 years of age (88.2%) and had at least some college education (90.3%). Of the 1,000 nonsmokers, 38.3% were former smokers. One-fifth (21.9%) of the smokers smoked more than a pack of cigarettes per day (>20 cigarettes). 

Knowledge and beliefs about cigarette litter are presented in Table 2. Data are presented for the entire sample broken out by smokers and nonsmokers. Taken together, most respondents (71.0%) correctly identified cigarette filters as the most collected item during beach clean-ups. The majority of respondents also believe that cigarette butts are toxic (75.8%), agree that it can be harmful to dispose of cigarette butts in a trashcan (84.3%), and consider cigarette butts to be litter (91.8%). Additionally, the majority of respondents disagreed or don’t know that cigarette butts are biodegradable (82.4%) and harmless when eaten by humans (90.9%) or marine life (90.7%). 

**Table 2:** Attitudes and beliefs about cigarette butts as litter (n = 1,000 smokers & 1,000 nonsmokers).

	Smokers		Nonsmokers			
**The single most collected item in beach waste cleanup each year is:**	**n**	**%**	**n**	**%**	**χ^2^**	***P***
Beverage containers	164	16.4	190	19.0	9.45	0.02
Cigarette filters (butts)	739	73.9	680	68.0		
Fishing line/nets	7	0.7	12	1.2		
Plastic bags	90	9.0	118	11.8		
**Cigarette butts are toxic**						
Strongly Disagree/Disagree/DK *	277	27.7	207	20.7	13.36	0.00
Strongly Agree/Agree	723	72.3	793	79.3		
**Cigarette butts are biodegradable**						
Strongly Disagree/Disagree/DK	789	78.9	858	85.8	16.38	0.00
Strongly Agree/ Agree	211	21.1	142	14.2		
**Cigarette butts are harmless when eaten by humans**						
Strongly Disagree/Disagree/DK	884	88.4	934	93.4	15.11	0.00
Strongly Agree/Agree	116	11.6	66	6.6		
**Cigarette butts are harmless when eaten by animals/marine life**						
Strongly Disagree/Disagree/DK	871	87.1	942	94.2	29.74	0.00
Strongly Agree/Agree	129	12.9	58	5.8		
**It can be dangerous to throw a cigarette butt in a trashcan**						
Strongly Disagree/Disagree/DK	178	17.8	136	13.6	6.66	0.01
Strongly Agree/Agree	822	82.2	864	86.4		
**Do you consider cigarette butts to be litter?**						
No/DK	138	13.8	27	2.7	81.39	0.00
Yes	862	86.2	973	97.3		

* DK = “don’t know”.

Prevalence of cigarette butt littering, both ever littering and past-month littering, is shown in Table 3. Almost three-quarters (74.1%) of smokers reported littering their cigarette butts at one point in their lifetime by disposing of them on the ground or throwing them out of a car window. When asked about littering cigarette butts in the past month, 55.7% reported either disposing of cigarette butts on the ground, in a sewer/gutter, or down a drain. 

**Table 3:** Past-month **^†^** and ever littering * of cigarette butts (n = 1,000, smokers only).

Littered cigarette butts in past month	n	%
No	443	44.3
Yes	557	55.7
**Ever littered cigarette butts**		
No	259	25.9
Yes	741	74.1

^† ^Past-month littering = Disposing of cigarette butts on the ground, in a sewer/gutter, or down a drain in the last 30 days. * Ever littering = ever disposing of cigarette butts by dropping them on the ground or throwing/dropping them out of a car window.

Table 4 displays the bivariate and multivariate findings for ever littering. Two of the independent variables were significantly related to ever littering in the bivariate analyses: the belief that cigarette butts are biodegradable (OR = 1.47, 95%CI = 1.02, 2.13), and the belief that cigarette butts are litter (OR = 3.83, 95%CI = 2.13, 6.93). Only the latter variable remained statistically significant in the multivariate model (OR = 3.68, 95%CI = 2.04, 6.66), which included only those variables significant in the bivariate analysis. Those who did not believe or were not sure whether cigarette butts are litter were over three and half times as likely to report having littered their cigarette butts on the ground or out of a car window at one point in their lifetime.

**Table 4:** Statistically significant predictors of ever littering **^†^** cigarette butts among smokers (n = 1,000).

	Bivariate		Multivariate *	
	OR	95%CI	OR	95%CI
**Cigarette butts are biodegradable**				
Strongly Disagree/Disagree/DK	Ref		Ref	
Strongly Agree/ Agree	**1.47**	**(1.02, 2.13)**	1.32	(0.91, 1.92)
**Do you consider cigarette butts to be litter?**				
No/DK	**3.83**	**(2.13, 6.93)**	**3.68**	**(2.04, 6.66)**
Yes	Ref		Ref	

^† ^Ever littering = ever disposing of cigarette butts by dropping them on the ground or throwing/dropping them out of a car window. * The multivariate model includes only those significant variables in bivariate analysis. OR = Odds ratio; Ref = referent.

Table 5 displays the bivariate and multivariate findings for past-month littering. Four of the independent variables were significantly related to past-month littering in the bivariate analyses: male gender (OR = 1.54, 95%CI = 1.19. 1.99); black or African American race (OR = 1.78, 95%CI = 1.06, 2.97); the belief that cigarette butts are biodegradable (OR = 1.58, 95%CI = 1.16, 2.17); and the belief that cigarette butts are not litter (OR = 4.26, 95%CI = 2.70, 6.70). 

In a multivariate logistic regression containing the variables significant in the bivariate analysis, however, only male gender (OR = 1.49, 95%CI = 1.14, 1.94) and the belief that cigarette butts are not litter (OR = 4.00, 95%CI = 2.53, 6.32) remained statistically significantly related to littering cigarette butts in the past month. Those who did not believe that cigarette butts are litter were four times as likely to have littered cigarette butts on the ground, in a gutter, or down a drain in the past month. 

**Table 5:** Statistically significant predictors of past-month cigarette butt littering ^†^ among smokers (n = 1,000).

	Bivariate		Multivariate *	
	OR	95%CI	OR	95%CI
**Gender**				
Male	**1.54**	**(1.19, 1.99)**	**1.49**	**(1.14, 1.94)**
Female	Ref		Ref	
**Race**				
White	Ref		Ref	
Black/AA	**1.78**	**(1.06, 2.97)**	1.69	(1.00, 2.87)
Asian or PI	1.20	(0.68, 2.11)	0.99	(0.55, 1.78)
NA/AN	1.06	(0.28, 3.99)	0.98	(0.24, 3.94)
Mixed Race	1.49	(0.72, 3.07)	1.85	(0.88, 3.86)
Declined	1.20	(0.57, 2.56)	1.02	(0.47, 2.23)
**Cigarette butts are biodegradable**				
Strongly Disagree/Disagree/DK	Ref		Ref	
Strongly Agree/ Agree	**1.58**	**(1.16, 2.17)**	1.31	(0.95, 1.83)
**Do you consider cigarette butts to be litter?**				
No/DK	**4.26**	**(2.70, 6.70)**	**4.00**	**(2.53, 6.32)**
Yes	Ref		Ref	

* The multivariate model includes only those significant variables in bivariate analysis. OR = Odds ratio; Ref = referent. ^† ^Past-month littering = Disposing of cigarette butts on the ground, in a sewer/gutter, or down a drain within the last 30 days.

When all independent variables were included in the models, not just those which were significant in the bivariate analyses, results were the same, with one exception: black/African American race remained statistically significant in the past-month analysis. Models for both outcome variables, ever littering and past-month littering, were refined using stepwise logistic regression, with unchanging results.

## 4. Discussion

The majority of respondents believed that cigarette butts are harmful to the environment. However, one quarter of respondents did not believe cigarette butts are toxic, and more nonsmokers believed cigarette butts to be litter than smokers. In fact, across all cigarette litter attitude and belief items, nonsmokers endorsed the attitude that cigarette butts are harmful to the environment in greater proportions than smokers, indicating that more education needs to be done to address this dichotomy. Even though most smokers acknowledged that littering tobacco products could have damaging effects, our study found a clear disconnect between behaviors and beliefs. Despite the fact that 86% of smokers consider cigarette butts litter, three-quarters reported disposing of them on the ground or out of a car window at one time or another. 

To our knowledge, this was the first national non-industry-sponsored study of cigarette littering knowledge, beliefs and practices. In Keep America Beautiful’s 2009 “Littering Behavior in America” report funded by Philip Morris, researchers observed individuals’ littering behavior in 130 public outdoor locations, and found that among all instances when smokers discarded cigarettes, 65% were littered [28,35]. Although Keep America Beautiful measured observed littering (rather than reported littering), their rate is comparable to the self-reported past-month and ever-littering rates found in this study. Keep America Beautiful also measured littering intentions and found that smokers were most likely to report that, among all types of items, they would most likely litter cigarette butts [28]. 

While many states have conducted observational litter studies, only a few have surveyed their residents about their self-reported tobacco littering behaviors. However, a 2001 report of littering in Iowa found that 18% of respondents had littered tobacco products in the past two years [36] and a 2009 study sponsored by the Texas Department of Transportation found a 6% past-month cigarette littering rate [37]. While Iowa and Texas’ rates appear lower than those found in our study, it is difficult to compare their findings to this study because the state analyses include a large proportion of nonsmokers. The same study of Texas drivers found that 62% of smokers said they or their passengers have at some point thrown a cigarette butt out of the car window [37]. 

Other countries have conducted national cigarette littering studies. In accordance with our findings, a 1996 nationally representative study by Tidy Britain Group (formerly ENCAMS, now Keep Britain Tidy) found that 75% of smokers in the UK had ever dropped their cigarette butts on the ground. As in the current study, the UK study also found that men are more likely than women to litter their cigarettes [38]. Another industry estimate from a Philip Morris-sponsored study in 1995 indicated that 45% of smokers disposed of their cigarette filters on the ground when attending events outside [39].

Regression analyses in this study showed that not considering cigarette butts to be litter was the primary factor that influenced cigarette littering behavior. The Texas Department of Transportation study also found that 8% of smokers who have thrown cigarettes out of their cars do not consider cigarettes butts litter compared to almost 13% of smokers who littered in this study [37]. However, comparing our results to Texas’ is problematic since their smoker sample was very small. 

In a qualitative study by one tobacco manufacturer from the mid-1990s, researchers found that the perception of cigarettes as litter depended on the quantity of cigarette filters on the ground. The greater the number of cigarettes in the picture respondents were shown, the more likely they were to perceive the used cigarettes as litter [39]. Similarly, a 2008 ENCAMS qualitative study found that smokers only felt that littered cigarette butts were a problem when there were several in sight [40]. Focus groups conducted by Philip Morris in 1998 found that adult smokers acknowledged their behavior as littering but justified it as acceptable since there did not appear to be an environmental impact [41]. Washington state conducted litter focus groups in 2000 and concluded that “clearly defining what constitutes litter” should be considered and cited cigarette litter as a major example [42]. Based on our findings and previous research, cigarette litter prevention campaigns that emphasize that cigarette butts—in even the smallest quantities—are indeed litter could have an impact on behavior. Yet, given that most smokers in our sample acknowledge that cigarette butts are litter yet litter them anyway, the term “litter”, is, perhaps, too innocuous to change behavior. In this study, the authors refer to “cigarette litter” as the term to describe improperly discarded cigarette butts for the purpose of our survey. However, other cigarette litter clean up advocates, such as the Cigarette Butt Pollution Project (CBPP) [43] led by Dr. Thomas Novotny, the primary advocate and researcher in the U.S. on this issue, has reframed the problem as “cigarette butt waste” rather than “litter.” This difference in terminology is recommended on the CBPP website, stating that “Butt waste isn’t just litter: Filters falsely reassure smokers, and cigarette waste damages habitat, landscapes, and ecosystems; ignites destructive, deadly fires; poisons wildlife and children; consumes tax dollars for cleanup and disposal; and lasts forever!” [44]. According to Dr. Novotny, since many U.S. states treat electronics and plastic bottles as toxic waste through various policies, in future discourse, consciously emphasizing the true environmental threat of cigarette butts as waste could ignite similar public concern [9]. 

While only significant in bivariate analyses, belief that cigarettes are biodegradable did approach significance in predicting cigarette littering behavior. Past research into tobacco industry documents shows that the industry has conducted focus groups with smokers on this issue [12]. A qualitative study by Philip Morris in 1995 found that most smokers do perceive used cigarette filters to be biodegradable [39]. The 2008 ENCAMS study found that smokers perceived cigarette butts as “biodegradable and easily displaced” [40]. On the other hand, results of focus groups conducted in 1997 on behalf of Philip Morris found that, in concurrence with our findings, most smokers did *not* believe cigarette filters to be biodegradable [45]. This confusion reflects the need for a clear message about cigarette filter biodegradability to the public. The message should address this misunderstanding by clarifying that although filters are technically biodegradable, in reality, they do not disappear from places where people actually drop them. Alternatively, perhaps a more convincing and concise argument to the public is that regardless of whether or not the plastic filter breaks down, toxic chemicals released from cigarette butts can persist in water and soil.

Research on cigarette litter attitudes and behavior is vital to crafting communication campaigns that will shift social norms. Understanding why smokers toss their cigarettes to the ground or out of their car windows can inform not only anti-littering messaging, but perhaps quit-smoking messages tailored for environmentalists as well. The harmful impact that the ubiquitous cigarette butt has on the environment could be another reason for smokers to quit. In fact, public health campaigns have recently started to promote this message alongside information about the health and economic impacts of smoking. In their 2011 “Thrown Away” advertisement, the California Tobacco Control Program shows a cigarette butt on its journey from a smoker’s mouth to the pavement, through the sewer and finally landing on a beach, warning viewers that “cigarettes are not just dangerous when they’re smoked. They’re dangerous long after” [46]. 

## 5. Limitations

The ever-littering and past-month littering questions were worded differently such that they measured different littering behaviors, not just different time frames. Had the past-month littering item been worded the same as the ever-littering item, *i.e.*, have you ever discarded your cigarette on the ground or out of a car window, it is possible that the proportion of smokers indicating they had littered in the past month would have been different. 

In addition, littering is a sensitive behavior. As such, it is possible that self-report of littering behavior is underestimated in this study, making our estimate of cigarette littering prevalence a conservative one. For instance, our regression analyses found that among smokers, men were more likely to report that they had tossed their cigarette butts down a drain, in a gutter or sewer or on the ground in the past month compared to women. However, Keep America Beautiful reported that when observing behavior, men and women were equally likely to litter their cigarette butts. They concluded that, across all types of littering, females and males littered in equal proportions but that men were more likely to *report* their behavior and felt a lower personal obligation to not litter [28]. Given these findings, it is not surprising that in ENCAMS’ research, they found that women felt guiltier about littering cigarette butts and some even characterized the action as more improper for women [40]. An anti-cigarette littering campaign might be successful if it targets women and perhaps, exposes the hypocrisy of people who claim they do not litter. 

Also, while the lack of peer-reviewed literature on cigarette litter attitudes and behavior made item creation necessary, the investigator-created survey questions were not tested for validity or reliability prior to implementation. Lastly, the authors are limited in the data available with which to compare to the results of this study. The vast majority of national litter studies with data specifically on cigarette waste attitudes were directed or supported by the tobacco industry. Thus, instead of not comparing the data to published studies, it was decided to compare the findings of this study with the only available national data, which is industry sponsored.

## 6. Conclusions

While most smokers and nonsmokers today understand that cigarette litter is an environmental problem, a minority of smokers still do not recognize flicked cigarettes butts as litter or waste. We found that this misunderstanding was associated with littering behavior, indicating that education is still needed, especially in light of new evidence on the toxicity of cigarette butts [14,15]. This study begins to fill an important gap in the literature on non-tobacco industry funded research on tobacco littering. Our findings could help to inform a cessation campaign tailored for environmentalists whose message is to encourage smokers to reduce the damage that cigarettes have done to both their health and to the global environment. Future research could examine whether an environmental message is effective in smoking cessation. 
